# Maximum Entropy Criterion for Moment Indeterminacy of Probability Densities

**DOI:** 10.3390/e26020121

**Published:** 2024-01-30

**Authors:** Jordan M. Stoyanov, Aldo Tagliani, Pier Luigi Novi Inverardi

**Affiliations:** 1 Institute of Mathematics & Informatics, Bulgarian Academy of Sciences, 1113 Sofia, Bulgaria; 2 Faculty of Mathematical Sciences, Shandong University, Jinan 250100, China; 3 Department of Economics & Management, University of Trento, 38100 Trento, Italy; aldo.tagliani@unitn.it (A.T.); pierluigi.noviinverardi@unitn.it (P.L.N.I.)

**Keywords:** probability density, moments, Stieltjes and Hamburger moment problems, Hankel matrices, determinacy, indeterminacy, maximum entropy, MaxEnt criterion for M-indeterminacy, 44A60, 60E05, 62E10, 94A17

## Abstract

We deal with absolutely continuous probability distributions with finite all-positive integer-order moments. It is well known that any such distribution is either uniquely determined by its moments (M-determinate), or it is non-unique (M-indeterminate). In this paper, we follow the maximum entropy approach and establish a new criterion for the M-indeterminacy of distributions on the positive half-line (Stieltjes case). Useful corollaries are derived for M-indeterminate distributions on the whole real line (Hamburger case). We show how the maximum entropy is related to the symmetry property and the M-indeterminacy.

## 1. Introduction

When studying probability distributions, one of the challenging questions we arrive at comes from the classical moment problem. The question is whether or not a probability distribution is uniquely determined by the sequence of all moments, assuming they are finite. The answer can be given for the distribution itself; equivalently, for the associated random variable *X*; its distribution function *F*; the density $f=F^{\prime }$; or the bounded positive measure $\mu =\mu_{F}$ induced by *F*. Thus, if the answer is positive, we call the distribution (also X,F,f,μ) M-determinate; otherwise, we call it M-indeterminate. (Here, ‘M’ stands for ‘Moment’.)

It is well known that if μ is M-indeterminate, then there are infinitely many absolutely continuous distributions, infinitely many purely discrete distributions, and infinitely many singular distributions, all having the same moments as μ.

It is important, from both the theoretical and applied points of view, to have criteria at hand allowing to specify/identify the determinacy or indeterminacy property of a distribution. The best is to work with conditions which are in the group ‘checkable conditions’; comments and references are given at the end of our paper. There is another group of ‘non-checkable conditions’. Here are the well-known classical necessary and sufficient conditions for the (in)determinacy of μ in terms of the limits of the smallest eigenvalues of sequences of Hankel matrices. Our recent review paper [1] describes the whole spectrum, called ‘a bunch’, of the fundamental results, old and recent. The reader will find in [1] details about the great contributions of T. Stieltjes, H. Hamburger, N. Akhiezer, M. Krein, C. Berg, K. Schmüdgen, M. Putinar, B. Simon, and others. Their works are widely known.

Developments over the last few decades have shown the efficiency of involving the *Principle of Maximum Entropy*, see, for example, [2,3,4]. We also use the terms ‘maximum entropy approach’ and ‘maximum entropy method’. For ‘maximum entropy’, we write the traditional ‘MaxEnt’.

The idea of the MaxEnt method consists in selecting a distribution which possesses maximum uncertainty, and at the same time, fulfills the restrictions imposed by the known information.

In general, it is more delicate to deal with M-indeterminate distributions, since we need, for example, to know how to find, describe and work with an infinite family of distributions all having the same moments. In any case, MaxEnt may help to shed light on this ‘dark tunnel’.

In this paper, we follow the generally accepted terminology and notations as used in probability theory. We write X∼F for a random variable *X* whose distribution function is *F*, with $f=F^{\prime }$ being the density, and specify the range U of values of *X*, the support of *F*, which is assumed to be unbounded. Only in this case can the ‘interesting’ property of M-indeterminacy appear. We work with the moment sequence ${\left\{m_{k}\right\}}_{k=0}^{\infty }$, and if U=R=(−∞,∞), this is a Hamburger case, while with $U=R_{+}=\left[0,\infty \right),$ it is a Stieltjes case.

For *X* being an absolutely continuous random variable with strictly positive density *f*, we are looking for conditions, or criteria, guaranteeing the M-determinacy or M-indeterminacy of μ. We use the entropy (called also ‘differential entropy’), which is denoted by $h_{f}$ and defined as follows:

$$h_{f}:=E\left[-\ln f\left(X\right)\right]=-\int_{U}\left(\ln f\left(x\right)\right)\;f\left(x\right)\;dx.$$

The idea is very natural: We start with the *n*-truncated moment set ${\left\{m_{k}\right\}}_{k=0}^{n},$ and based on it, we find the MaxEnt approximant $f_{n}$ of *f* and study the limit of the entropy $h_{f_{n}}$ of $f_{n}$ as n→∞.

There is a remarkable fact, namely, that there are only two possibilities for the ‘value’ of the limit $\lim_{n\to \infty }h_{f_{n}}$; either it is a finite number, or it is ‘equal’ to −∞. Depending on this limit, we decide that *f* is M-determinate or M-indeterminate.

It is relevant to mention one of the results proved in ([5], Theorem 1): if an absolutely continuous distribution *F* with density *f* is M-determinate, then the sequence of MaxEnt approximants is converging in entropy to *f*. One of our goals in this paper is to involve additional arguments allowing to show that such a result on entropy convergence can be extended to the case of M-indeterminate distributions.

The remainder of the paper is organized as follows. In Section 2, we recall briefly what we need about Hankel matrices and introduce the MaxEnt setup. In Section 3, we calculate the entropy of densities with the given *n*-truncated moment set, for fixed *n*, and also for the entire moment sequence ${\left\{m_{k}\right\}}_{k=0}^{\infty }.$ In Section 4, we provide an M-indeterminacy MaxEnt criterion in the Stieltjes case. In Section 5, we present corollaries related to the M-indeterminacy in the Hamburger case. Discussed is the question: Among a family of infinitely many densities all with the same moments, which density has the largest entropy?

## 2. Basics of Hankel Matrices and the MaxEnt Setup

When we tell that ${\left\{m_{k}\right\}}_{k=0}^{\infty }$ with $m_{0}=1$ is a moment sequence, it always means that there is a probability measure $\mu =\mu_{F}$ which is ‘behind’. Thus, think of a random variable *X* defined in an underlying probability space (Ω,F,P), taking values in a set U⊂R. If *F* is its distribution function, F(x):=P[X≤x],x∈R, then $\mu =\mu_{F}$ is a positive Borel measure induced by *F*. We write just μ.

A basic assumption is that $E[|X{|}^{k}]<\infty$ for all k=1,2,…. Thus, well defined are the moments

$$m_{k}=E\left[X^{k}\right]=\int_{\Omega }X^{k}\left(\omega \right)\;dP=\int_{U}x^{k}\;dF\left(x\right)=\int_{U}x^{k}f\left(x\right)\;dx=\int_{U}x^{k}\;\mu \left(dx\right),\;k=1,2,\ldots ,$$

and also the moment sequence ${\left\{m_{k}\right\}}_{k=0}^{\infty }.$ If U=R, we say that ${\left\{m_{k}\right\}}_{k=0}^{\infty }$ is a *Hamburger moment sequence*, while for $U=R_{+}$, ${\left\{m_{k}\right\}}_{k=0}^{\infty }$ is a *Stieltjes moment sequence.*

For any moment sequence ${\left\{m_{k}\right\}}_{k=0}^{\infty }$, we define a few infinite sequences of *Hankel matrices*, namely, ${\left\{H_{n}\right\}}_{n=1}^{\infty }$ and ${\left\{H_{n,p}\right\}}_{n=1}^{\infty }$, and their *determinants*, as follows:

$$H_{n,p}={\left(m_{i+j+p}\right)}_{i,j=0}^{n},\;D_{n,p}:=\det\;\left(H_{n,p}\right),\;p=0\;\mathrm{or}\;1.$$

If p=0, $H_{n}:=H_{n,0}$ is the ‘basic’ Hankel matrix, $H_{n,p},$ for p=1 is the ‘shifted’ Hankel matrix: $H_{n,1}={\left(m_{i+j+1}\right)}_{i,j=0}^{n}$ is based on the ‘shifted’ moment sequence $\left\{m_{1},m_{2},\ldots \right\}$ generated by the measure $\mu_{1}$ with $d\mu_{1}=xd\mu$.

In what follows, we involve and use the entropy of the strictly positive density *f* under the constraint of knowing only the *n*-truncated moment set ${\left\{m_{k}\right\}}_{k=0}^{n}.$ We will see that the MaxEnt formalism allows to study in parallel both the Hamburger and the Stieltjes cases; hence, we assume that the distributions and their densities have support U=R or $U=R_{+}$.

Let us consider the Stieltjes case. For a density *f* with *n*-truncated moment set ${\left\{m_{k}\right\}}_{k=0}^{n},$ there is a density, say $f_{n},$ satisfying two properties:

(a) The ‘first’ *n* moments of $f_{n}$ are exactly ${\left\{m_{k}\right\}}_{k=0}^{n};$
(b) $f_{n}$ maximizes the Shannon entropy.

It is well known, see [2], that

$$f_{n}\left(x\right)=\exp\left(-\sum_{j=0}^{n}\lambda_{j}x^{j}\right),\;x\in R_{+},$$

where $\left(\lambda_{0},...,\lambda_{n}\right)$ are the Lagrange multipliers satisfying the constraints

$$\int_{R_{+}}x^{j}f_{n}\left(x\right)\;dx=m_{j},\;j=0,...,n.$$

In this case, we use the simple notation $h_{f_{n}}$ for the entropy of $f_{n},$ and remember that $h_{f_{n}}$ depends on the moments ${\left\{m_{k}\right\}}_{k=0}^{n}$. It is easy to see that

$$h_{f_{n}}=-\int_{R_{+}}\left(\ln f_{n}\left(x\right)\right)f_{n}\left(x\right)\;dx=\sum_{j=0}^{n}\lambda_{j}m_{j}.$$

We would like the sequence of approximants $\left\{f_{n}\right\}$ and the entropy sequence $\left\{h_{f_{n}}\right\}$ to be well defined for any n=1,2,…. It may happen, see ([6], Theorem 1), that for given *f* and ${\left\{m_{k}\right\}}_{k=0}^{n}$, the desired density $f_{n}$ does not exist, in which case the quantity $h_{f_{n}}$ is meaningless. However, in the cited paper, the following useful relation is established (the class $D_{n}$ is defined at the end of this section): $\sup_{f\in D_{n}}h_{f}=h_{f_{n-1}}$, even if the MaxEnt approach does not apply. Since the entropy is monotone and non-increasing as *n* increases, the latter equality enables us to simply set $h_{f_{n}}=h_{f_{n-1}}$, thus filling the ‘gap’ left by the non-existing densities $f_{n}$. This justifies the assumption made in the sequel, without loss of generality, that all entries of the monotone non-increasing entropy sequence ${\left\{h_{f_{n}}\right\}}_{n=1}^{\infty }$ are well defined.

In the non-symmetric Hamburger case, once the *n*-truncated moment set ${\left\{m_{k}\right\}}_{k=0}^{n}$ for even *n* is assigned, the positivity of the Hankel determinant $D_{n,0}=D_{n,0}\left(m_{0},\ldots ,m_{n}\right)$ guarantees the existence of a MaxEnt solution, see ([6], Appendix A). As a consequence, the entire entropy sequence ${\left\{h_{f_{n}}\right\}}_{n=1}^{\infty }$ is defined. In the symmetric Hamburger problem, the MaxEnt density existence is guaranteed under conditions similar to those in the Stieltjes case.

Now suppose that ${\left\{m_{k}\right\}}_{k=0}^{n+1}$ is a moment set for which we ‘keep fixed’ (unchanged) the moments $\{m_{0},m_{1},\cdots ,m_{n}\},$ while we treat as ‘varying continuously’ the moment $m_{n+1}$. If letting $b:=m_{n+1}$, then the (n+1)-truncated moment set can be written as $\left\{m_{0},m_{1},\ldots ,m_{n},b\right\}$. Moreover, the existence conditions for a solution of the moment problem require the Hankel determinants to be positive. This is guaranteed by imposing *b* to have a lower bound, say $b_{p,n+1}^{-}$, which is the unique real number satisfying the equation

$$D_{n,p}\left(m_{p},\ldots ,m_{n},b_{p,n+1}^{-}\right)=0,\;\mathrm{for}\;p=0\;\mathrm{or}\;1.$$

As well, due to the MaxEnt machinery, see ([6], Appendix A), in the Stieltjes case, the following value of *b* has to be considered:

$$b_{n+1}^{+}=\int_{R_{+}}x^{n+1}f_{n}\left(x\right)\;dx.$$

Notice that in general, $b_{n+1}^{+}\neq m_{n+1}$. Recall that we deal with the truncated (n+1)-moment set in which the parameter *b* stands for the (n+1)th moment:

$$\left\{m_{0},m_{1},\ldots ,m_{n},b\right\},\;\mathrm{where}\;b_{p,n+1}^{-}\leq b\leq b_{n+1}^{+}\;\mathrm{for}\;p=0\;or\;1.$$

In the Stieltjes case, we introduce the following classes of densities:

$$D_{n}:=\left\{f>0\;:\;\int_{R_{+}}x^{j}f\left(x\right)\;dx=m_{j},\;j=1,\ldots ,n\right\},$$

$$D_{\infty }:=\left\{f>0\;:\;\int_{R_{+}}x^{j}f\left(x\right)\;dx=m_{j},\;j=1,2,\ldots \right\}.$$

Similar notions can be introduced also in the Hamburger case, just replacing $R_{+}$ with R.

The class $D_{n}$ is a convex set for each *n*, and then $D_{\infty }={⋂}_{1}^{\infty }D_{n}$ is also convex. We know that in the M-indeterminate case, $D_{\infty }$ contains ‘infinitely many’ densities, all being solutions of the same moment problem.

For both the Hamburger and Stieltjes cases, we need to recall a few known facts which will be essentially used later.

***Fact*** ***1.*** We are going to work the moment sequence ${\left\{m_{k}\right\}}_{k=0}^{\infty }$ whose underlying density *f* has entropy $h_{f}$ such that either $h_{f}$ is finite, or $h_{f}=-\infty$. To be precise, distributions with $h_{f}=+\infty$ are not allowed. The reason for this is that once ${\left\{m_{k}\right\}}_{k=0}^{\infty }$ is assigned, the ‘option’ $h_{f}=+\infty$ is not feasible since it is well known in the MaxEnt setup that $h_{f}\leq h_{f_{2}}$, where $h_{f_{2}}=\frac{1}{2}\ln\left[2\pi \;e\;\left(m_{2}-{\left(m_{1}\right)}^{2}\right)\right]$ is finite because of Lyapunov’s inequality $m_{2}-{\left(m_{1}\right)}^{2}\geq 0$ (Hamburger case) and $h_{f}\leq h_{f_{1}}=1+\ln m_{1}$ is finite for every $m_{1}>0$ (Stieltjes case).

***Fact*** ***2.*** Once the moment set ${\left\{m_{k}\right\}}_{k=0}^{n}$ is given and $f_{n}$ is the corresponding MaxEnt density, the entropy sequence ${\left\{h_{f_{n}}\right\}}_{n=1}^{\infty }$ is monotone non-increasing, and its limit is either finite or −∞.

***Fact*** ***3.*** For consistency between the differential entropy of a continuous random variable and the entropy of its discretization, the differential entropy of any discrete measure which can be compared with Dirac’s deltas set is assumed to be −∞, see ([3], pp. 247–249).

***Fact*** ***4.*** If the density *f* is bounded, this is sufficient to eliminate the option $h_{f}=-\infty$. Indeed, suppose that 0<f(x)≤L<∞ for all *x*. It follows that

$$-h_{f}=\int_{U}\left(\ln f\left(x\right)\right)f\left(x\right)\;dx<\int_{U}\left(\ln L\right)f\left(x\right)\;dx=\ln L\;\Rightarrow \;h_{f}\geq -\ln L>-\infty .$$

## 3. Entropy of Densities Which Are M-Indeterminate

The MaxEnt formalism allows to treat both Hamburger and Stieltjes cases in a similar way. For the sake of brevity, we confine ourselves mainly to discussions on the Stieltjes case. All arguments can then be easily extended to the Hamburger case. This possibility is one of the advantages of involving the MaxEnt machinery.

### 3.1. Entropy of Densities from the Class $D_{n}$

We start with the formulation and the proof of the following result.

**Theorem** **1.** *Suppose that ${\left\{m_{k}\right\}}_{k=0}^{\infty },m_{0}=1,$ is the full moment sequence of a given density f. For fixed n, based on the n-truncated moment set ${\left\{m_{k}\right\}}_{k=0}^{n},$ we consider $f_{n},$ the* MaxEnt *approximant of f, and let $h_{f_{n}}$ be the entropy of $f_{n}$. Then, there are infinitely many densities $g\in D_{n}$ whose entropy $h_{g}$ is spanning an interval, namely*

(1) $$h_{g}\;\in \;\left(-\infty ,\;h_{f_{n}}\right].$$

**Proof.**  We provide arguments in both cases, Stieltjes and Hamburger.

(a) *Stieltjes case*. Preliminarily, for fixed n, let us consider $f_{n}$ and the upper bound $b_{n+1}^{+}$ of its (n+1)th order moment. It was mentioned that, in general, $b_{n+1}^{+}$ is different from $m_{n+1}$. Our goal is to specify the range of values of the entropy $h_{g}$, where *g* is an arbitrary density from the class $D_{n}$. For this, we introduce the following suitable subclass $E_{n}\subset D_{n}:$

$$E_{n}=\left\{f_{n+1}=\left(f_{n+1}\;|\;\mathrm{fixed}\;m_{1},\ldots ,m_{n},b\right)\},\;\mathrm{where}\;b\in \left(b_{p,n+1}^{-},b_{n+1}^{+}\right]\right\},\;p=0\;\mathrm{or}\;1.$$

Notice that we rely here on the specific truncated moment set $\left(m_{0},m_{1},...,m_{n},b_{p,n+1}^{-}\right)\in \partial \left(m\left(D_{n+1}\right)\right)$, the boundary of the moment space. Equivalently, the elements of $E_{n}$ are MaxEnt densities which are constrained by $\left(m_{1},\cdots ,m_{n},b\right)$; they belong to $D_{n}$ and, primarily, they all have analytically tractable entropy. The latter property enables us to calculate the entropy of all $g\in D_{n}$ by evaluating the entropy of all $g\in E_{n}.$

Let us consider $f_{n+1}$ for *b* varying in the interval $\left(b_{p,n+1}^{-},b_{n+1}^{+}\right]$ and calculate the values spanned by the entropy $h_{f_{n+1}}$.

*Subcase a1.* If $b=b_{n+1}^{+}$, the right-end point, it is easy to verify that $f_{n+1}$ has a Lagrange multiplier $\lambda_{n+1}=0$ and hence $f_{n+1}$ coincides with $f_{n}$; hence,

(2) $$h_{f_{n+1}}=h_{f_{n}}.$$

*Subcase a2.* If *b* is ‘close’ to the left-end point, i.e., $b\to b_{p,n+1}^{-}$, we look at the Hankel determinants $D_{n,0}$ and $D_{n,1}$ and see that either $D_{n,0}\to 0$ or $D_{n,1}\to 0$. This implies that the underlying measure μ is discrete; see, for example, ([7], Theorem 1.3, p. 6). Therefore, the entropy quantity $h_{f_{n+1}}$ is approaching −∞:

(3) $$\lim_{b\to b_{p,n+1}^{-}}h_{f_{n+1}}=-\infty .$$

It remains to mention an essential property of the entropy $h_{f_{n+1}}$ as a function of the variable *b*. Since $\frac{dh_{f_{n+1}}}{db}=\lambda_{n+1}>0,$ see ([2], Equation (2.73), p. 47), or the arguments below, we have that $h_{f_{n+1}}$ is monotone increasing with respect to $b\in (b_{p,n+1}^{-},b_{n+1}^{+}]$.

(b) *Hamburger case.* The arguments here are similar to those above. We need to replace $E_{n}$ by the following one with analogous meaning of all notations:

$${\tilde{E}}_{n}=\left\{f_{n+2}=\left(f_{n+2}\;|\;\mathrm{fixed}\;m_{1},\cdots ,m_{n},b_{n+1}^{+},b\right)\right\},\;\;\mathrm{where}\;\;b\in \left(b_{0,n+2}^{-},b_{n+2}^{+}\right].$$

Here, $b_{0,n+2}^{-}$ and $b_{n+2}^{+}$ are such that

$$D_{n,0}\left(m_{0},\ldots ,m_{n+1},b_{0,n+2}^{-}\right)=0,\;b_{n+q}^{+}=\int_{R}x^{n+q}f_{n}\left(x\right)\;dx,\;q=1,2.$$

In such a case, it is easy to see that

$$f_{n+2}=\left(f_{n+2}\;|\;\mathrm{fixed}\;m_{1},\cdots ,m_{n},b_{n+1}^{+},b_{n+2}^{+}\right)\;\equiv \;f_{n},$$

and hence, just as above, we conclude that $f_{n+2}$ satisfies the entropy relation $h_{f_{n+2}}=h_{f_{n}}$.

Joining together (2) and (3) (with obvious extension to the Hamburger case) with $f_{n+1}$ or $f_{n+2}$ and referring to the monotone increasing of the entropy with respect to *b*, we conclude that indeed there are infinitely many densities $f_{n+1}$ and $f_{n+2}\in E_{n}$ whose entropy spans the interval in (1), with this property holding for all $g\in D_{n}$. Theorem 1 is proved. □

### 3.2. Entropy of Densities from the Class $D_{\infty }$

Among the well-known properties of Shannon’s entropy, we use its concavity as a functional, which implies that the entropy of all densities $g\in D_{\infty }$ can be calculated.

We start with the Stieltjes moment sequence ${\left\{m_{k}\right\}}_{k=0}^{\infty }$ and calculate the entropy sequence ${\left\{h_{f_{n}}\right\}}_{n=1}^{\infty }$, which is monotone, non-increasing and convergent. Similarly, for a Hamburger moment sequence ${\left\{m_{k}\right\}}_{k=0}^{\infty }$, we calculate the entropy sequence ${\left\{h_{f_{n}}\right\}}_{n=2}^{\infty }$, being also monotone, non-increasing and convergent.

Let us show first that there exists only one density, say, $f^{*}\in D_{\infty }$ such that $f^{*}$ has the largest entropy, i.e.,

$$h_{f^{*}}=\max_{g\in D_{\infty }}h_{g}.$$

Indeed, the set of solutions to the S-indeterminate moment problem includes infinitely many densities, previously grouped in the convex set $D_{\infty }$. On the other hand, the continuous entropy functional $h:\;g\mapsto h_{g}=-\int_{U}\left(\ln g\left(x\right)\right)g\left(x\right)\;dx$ is strictly concave and, over the convex set $D_{\infty }$, $h_{g}$ attains its maximum value. Hence, we have that the optimization problem to maximize $h_{g}$ over $g\in D_{\infty }$ has indeed a unique solution $f^{*}$ such that $h_{f^{*}}:=\max_{g\in D_{\infty }}h_{g}.$

Relying on Theorem 1, we are ready to calculate the entropy of all moment equivalent densities $g\in D_{\infty }$. We keep in mind, all densities in the class $D_{\infty }$ have support $R_{+}$ in the Stieltjes case and R in the Hamburger case.

**Theorem** **2.** *Suppose that ${\left\{m_{k}\right\}}_{k=0}^{\infty }$ is the full Stieltjes moment sequence of a density f and it is known that f is M-indeterminate. We use $f_{n}$ and $h_{f_{n}}$ as before. Then, there are infinitely many densities $g\in D_{\infty }$ whose entropy $h_{g}$ is spanning an interval, namely,*

$$h_{g}\;\in \;\left(-\infty ,\;h_{*}\right],\;where\;h_{*}:=\underset{n}{\inf}h_{f_{n}}.$$

**Proof.**  Note first that each $g\in D_{\infty }$ satisfies $g\in {⋂}_{n=1}^{\infty }D_{n}$; hence, according to Theorem 1, *g* has entropy

$$h_{g}\in \overset{\infty }{\underset{n=1}{⋂}}\left(-\infty ,\;h_{f_{n}}\right]=\left(-\infty ,\;h_{*}\right].$$

This implies that

(4) $$h_{*}\geq h_{f^{*}}$$

which completes the proof. □

We use below, for example, S-determinate or H-determinate, meaning that a density is on $R_{+}$ or on R, and it is M-determinate or H-determinate. This similarly applies for S-indeterminacy and H-indeterminacy.

In general, it is not easy to establish the S-determinacy, and hence S-indeterminacy, through known criteria based on necessary and sufficient conditions. The existence of the density $f^{*}$ with the largest entropy, see Theorem 1, indicates that there is some similarity between the M-determinate and M-indeterminate cases. Consequently, since initially a finite set of moments is involved, the technique of density reconstruction through the MaxEnt approach can be applied without distinguishing these two cases.

With $f,\;\left\{m_{1},\ldots ,m_{n}\right\},\;f_{n},\;h_{f_{n}}$, all as above, we give here some details.

First, if *f* is S-determinate and H-determinate, the sequence of approximants ${\left\{f_{n}\right\}}_{n=1}^{\infty }$ converges in entropy to a unique underlying density *f*, see ([5], Section 3), that is, ${\left\{h_{f_{n}}\right\}}_{n=1}^{\infty }\to h_{f}$ as n→∞ with $\inf_{n}h_{f_{n}}$ all being finite. However, from the used procedure, relying on the geometrical meaning of Theorem 2.19, p. 72 in [7], it is immediate to deduce that the statement of convergence in entropy is equally extended to the case where $h_{f}=-\infty$, from which $\inf_{n}h_{f_{n}}=-\infty .$

Second, if *f* is S-indeterminate, the entropy sequence ${\left\{h_{f_{n}}\right\}}_{n=1}^{\infty }$ is monotone non-increasing and hence convergent with lower bound $h_{*}$, i.e.

$$h_{f_{n}}\;\to \;\underset{n}{\inf}h_{f_{n}}\;\geq \;h_{f^{*}}.$$

It is useful to mention that Theorem 2 and the comments completely agree with the rationale of the MaxEnt approach: when all known information has been taken into account, a system with maximum entropy is the most probable state because it is the system in which the least amount of information has been defined.

Moreover, Theorem 2 justifies the approach of reconstruction of the density *f*, starting from a finite set of moments and passing to the full moment sequence, regardless of the M-determinacy or M-indeterminacy of *f*. In any case, that issue is not really of great practical significance. In fact, a full set of moments will ‘never’ be available; hence, for practical purposes, we deal only with finite *n*, which is perhaps ‘big enough’. Nevertheless, $f_{n}$ is a valuable approximation of $f^{*}$ since both $f_{n}$ and $f^{*}$ have the same first *n* moments and $h_{f_{n}}>h_{f^{*}}.$ This fact also corresponds well to the MaxEnt rationale. Thus, the question of moment (in)determinacy of the density *f* is not essential for the procedure we follow.

## 4. Stieltjes Case: MaxEnt Criterion for M-Indeterminacy

We deal with a random variable *X* on $R_{+},\;X∼F,f=F^{\prime }$ with finite all moments ${\left\{m_{k}\right\}}_{k=1}^{\infty }$. Recall that $m_{0}=1.$ We mentioned in the Introduction one fundamental fact: if the distribution *F* is M-indeterminate, then there are infinitely many distributions of any kind, all having the same moments as *F*.

Recall that a Stieltjes moment sequence ${\left\{m_{k}\right\}}_{k=1}^{\infty }$ can also be considered a Hamburger moment sequence, i.e., it is coming from a random variable in R. We always have to make a distinction between M-determinacy and M-indeterminacy by specifying that it is in the sense of Stieltjes, or in the sense of Hamburger. We use below the obvious terms, S-determinate, S-indeterminate, H-determinate and H-indeterminate, in their short forms, S-det, S-indet, H-det and H-indet. Let us list the possibilities for the distribution *F*:

- If *F* is S-indet, it is also H-indet. If *F* is H-det, it is also S-det.
- If *F* is H-indet, then either *F* is also S-indet, or, it may look a little ‘surprising’, *F* is S-det.

Thus, we have three cases; they will be discussed below. Relying on the results in Section 3, we provide now a MaxEnt criterion for M-indeterminacy in the Stieltjes case.

**Theorem** **3.** (Main result.) *Let f be a probability density with finite all moments. Denote by $m:={\left\{m_{k}\right\}}_{k=1}^{\infty }$ its full moment sequence and $m_{n}:={\left\{m_{k}\right\}}_{k=1}^{n}$ its nth truncated set. If m is considered as a Stieltjes moment sequence, we write $f_{n}^{\left(S\right)}$ for the* MaxEnt *approximant of f based on $m_{n}.$ Similarly, $f_{n}^{\left(H\right)}$ will stand for the MaxEnt approximant of f based on $m_{n}$ if considering m as a Hamburger moment sequence. For the entropy, we use the notations $h_{f_{n}^{\left(S\right)}}$ and $h_{f_{n}^{\left(H\right)}}.$*

*The Stieltjes moment sequence ${\left\{m_{k}\right\}}_{k=1}^{\infty }$ corresponds to the moments of infinitely many distributions on $R_{+}$; equivalently, the distribution F is M-indeterminate, **if and only if** the following relation holds:*

$$\underset{n}{\inf}h_{f_{n}^{\left(H\right)}}>\underset{n}{\inf}h_{f_{n}^{\left(S\right)}}>-\infty .$$

**Proof.**  First, we recall the well-known result according to which if *n* is odd, the estimator $f_{n}^{\left(H\right)}$ does not exist. Since the entropy is monotone and non-increasing as *n* increases, it is proved in [6] (Section 3.2) that the entropy quantity $h_{f_{n-1}^{\left(H\right)}}$ can be associated with $f_{n}^{\left(H\right)}$ in the sense that $h_{f_{n}^{\left(H\right)}}=h_{f_{n-1}^{\left(H\right)}}$. Thus, the sequence $\left\{h_{f_{n}^{\left(H\right)}}\right\}$ will be well defined for each *n*, filling up the ‘initial’ gap left by the odd moments. Furthermore, the inequality $h_{f_{n}^{\left(H\right)}}>h_{f_{n}^{\left(S\right)}}$ is meaningful for any *n* since both $f_{n}^{\left(H\right)}$ and $f_{n}^{\left(S\right)}$ are based on the same constraints, the moment set ${\left\{m_{k}\right\}}_{k=1}^{n}$, whilst $f_{n}^{\left(S\right)}$ has an additional constraint, namely, the support is $U=R_{+}\subset R$.

Now we consider the three possibilities mentioned above. In brackets, we write what is *F* first, and what is second.

*Case 1.* [*F* is S-indet and H-indet] We refer to relation (4) from which it follows that $\inf_{n}h_{f_{n}^{\left(H\right)}}>\inf_{n}h_{f_{n}^{\left(S\right)}}>-\infty$ holds.

*Case 2.* [*F* is H-indet and S-det] We recall that the unique solution, a measure, with positive support is a Nevanlinna extremal solution whose spectrum contains 0 and is a discrete unbounded subset of [0,∞), see ([8], Remark 2.2.2, p. 178). Then, as quoted before, $\inf_{n}h_{f_{n}^{\left(H\right)}}$ is finite and $\inf_{n}h_{f_{n}^{\left(S\right)}}=-\infty .$

*Case 3.* [*F* is H-det and S-det] Clearly, one solution solely supported on [0,∞) exists. As a consequence, for the limit $L:=\inf_{n}h_{f_{n}^{\left(H\right)}}=\inf_{n}h_{f_{n}^{\left(S\right)}},$ we have that either *L* is finite or *L* is ‘equal’ to −∞. If *L* is finite, the distribution *F* is absolutely continuous with either bounded or unbounded density. If L=−∞, *F* is either absolutely continuous with unbounded density, or it is discrete.

It remains to show that the converse statements, call them Case 1c, Case 2c, and Case 3c, are also true. We show this by contradiction.

Indeed, in Case 1c, if $\inf_{n}h_{f_{n}^{\left(H\right)}}>\inf_{n}h_{f_{n}^{\left(S\right)}}>-\infty$ holds true, then both cases ‘H-indet with S-det’ and ‘H-det with S-det’ are not possible. This is because they respectively require both $\inf_{n}h_{f_{n}^{\left(S\right)}}=-\infty$ and $\inf_{n}h_{f_{n}^{\left(H\right)}}=\inf_{n}h_{f_{n}^{\left(S\right)}}$. These arguments show that *F* is H-indet and S-indet. The arguments to prove Case 2c and Case 3c are similar. □

It is worth mentioning that the criterion for M-indeterminacy established in Theorem 3, the Stieltjes case, cannot be extended to the Hamburger case. Indeed, from both Case 2 [H-indet with S-det] and Case 3 [H-det with S-det], the condition ‘finite lower bound $\inf_{n}h_{f_{n}^{\left(H\right)}}$’ does not distinguish the H-indeterminate case from the H-determinate case. Nevertheless, from Theorem 3, some useful corollaries concerning Stieltjes or Hamburger easily follow.

**Corollary** **1.** *A necessary condition for the distribution F to be S-indeterminate with H-indeterminacy is for both quantities $\inf_{n}h_{f_{n}^{\left(S\right)}}$ and $\inf_{n}h_{f_{n}^{\left(H\right)}}$ to be finite.*

**Corollary** **2.** *A sufficient condition for the distribution F to be H-determinate is that the quantity $\inf_{n}h_{f_{n}^{\left(H\right)}}$ is ‘equal’ to −∞.*

Notice that in the Stieltjes case, Theorem 3 provides also a sufficient condition to guarantee the existence of a density, which is equivalent to the absolute continuity property of the distribution *F*. Similarly, such a condition can be extended to the Hamburger case.

**Corollary** **3.** *Let ${\left\{m_{k}\right\}}_{k=0}^{\infty }$ be a strictly positive definite Stieltjes moment sequence which corresponds to the moments of exactly one distribution F. If $\inf_{n}h_{f_{n}^{\left(S\right)}}=\inf_{n}h_{f_{n}^{\left(H\right)}}$ is finite, then F is absolutely continuous with either bounded or unbounded density.*

## 5. Comments on M-Indeterminate Distributions on R

Here, we cite a result from ([9], Theorem 1 and Corollary 1, pp. 100–101); see also ([10], Examples 11.12 and 11.13) for a general family of distributions.

**General** **Statement.** *Suppose that F is a distribution function on R with moment sequence $\left\{m_{0},0,m_{2},0,m_{4},...\right\}$ (Hamburger case). Then, if F is M-indeterminate, symmetric and non-symmetric solutions exist.*

Besides the above sources, we can also refer to the notion *Stieltjes class*, S(f,h), introduced for any M-indeterminate distribution, see [11]. Recall that

$$S\left(f,h\right)=\{f_{\varepsilon }\left(x\right)=f\left(x\right)\left[1+\varepsilon p\left(x\right)\right],\;x\in R,\;\varepsilon \in \left[-1,1\right]\},$$

where $f=F^{\prime }$ is the density of the M-indeterminate distribution *F*, and p(x),x∈R, called a ‘perturbation function’, is a sign function with norm ||p||=1, satisfying the ‘vanishing moments’ property, $\int x^{k}f\left(x\right)p\left(x\right)\;dx=0,\;k=0,1,2,\ldots$ Another related recent work is [12].

It turns out that the MaxEnt technique enables us to make a further refinement of what we know about the symmetric solutions, also of the measures, which are M-indeterminate.

**Theorem** **4.** Suppose that F is an arbitrary distribution on R with finite moments and moment sequence $\left\{m_{0},0,m_{2},0,m_{4},...\right\}$ (Hamburger case) and that F is M-indeterminate. Then, the density $f^{*}$, see Section 3.2, with the largest entropy, is symmetric.

**Proof.**  Consider an arbitrary non-symmetric $g=\left(g\left(x\right),x\in R\right)\in D_{\infty }$. It is easy to verify that $\tilde{g}=\left(g\left(-x\right),x\in R\right)$ is such that $\tilde{g}\in D_{\infty }$. Moreover, *g* and $\tilde{g}$ have the same entropy, i.e., $h_{\tilde{g}}=h_{g}$. Consider the densities ${\tilde{g}}_{*}$ and $g_{*}$ for which the entropies $h_{\tilde{g}}$ and $h_{g}$ are maximal. They are both in the set $D_{\infty }$. Combining the above general statement with the uniqueness of the MaxEnt density $g_{*}$, it follows that ${\tilde{g}}_{*}\equiv g_{*}$; hence, $g_{*}$ is symmetric. □

## 6. Brief Conclusions

In this paper, we establish a new criterion for the M-indeterminacy of a probability density on the positive half-line (Stieltjes case) by involving the MaxEnt approach. Interesting corollaries are derived for probability densities on the whole real line (Hamburger case). The obtained results are new and they can be considered a valuable addition to the results based on two groups of conditions called ‘checkable’ or ‘uncheckable’ for either the M-determinacy or M-indeterminacy of distributions.

The recent review paper [1] contains a comprehensive description of significant results based on ‘uncheckable conditions’, including two illustrations of how to use this kind of condition as an indication for a specific property of a distribution in terms of its moments. However, from the applied point of view, most useful are the results involving ‘checkable conditions’. The reader is referred to the following sources: [10,13,14]. The property ‘M-indeterminacy’, besides its non-triviality as a mathematical phenomenon, arises in important applied areas. Among them are atmospheric studies, gravity theory and quantum mechanics, see, for example, [15,16,17]. The involvement of the MaxEnt technique may lead to challenging theoretical problems; however, the answers, when available, would shed additional light on the analysis of applied problems.

## Data Availability

No new data were created or analyzed in this study. Data sharing is not applicable to this article.
